# The influence of powered prostheses on user perspectives, metabolics, and activity: a randomized crossover trial

**DOI:** 10.1186/s12984-021-00842-2

**Published:** 2021-03-16

**Authors:** Jay Kim, Jeffrey Wensman, Natalie Colabianchi, Deanna H. Gates

**Affiliations:** 1 School of Kinesiology, University of Michigan, 401 Washtenaw Avenue, Ann Arbor, MI 48109-2214 USA; 2 Department of Mechanical Engineering, University of Michigan, Ann Arbor, MI USA; 3 University of Michigan Orthotics and Prosthetics Center, Ann Arbor, MI USA; 4 Department of Biomedical Engineering, University of Michigan, 401 Washtenaw Avenue, Ann Arbor, MI 48109-2214 USA

**Keywords:** Transtibial amputation, Microprocessor ankle, Inertial measurement unit, Accelerometer, Metabolic cost, Preference, Step count, Walking speed

## Abstract

**Background:**

Powered prosthetic ankles provide battery-powered mechanical push-off, with the aim of reducing the metabolic demands of walking for people with transtibial amputations. The efficacy of powered ankles has been shown in active, high functioning individuals with transtibial amputation, but is less clear in other populations. Additionally, it is unclear how use of a powered prosthesis influences everyday physical activity and mobility.

**Methods:**

Individuals with unilateral transtibial amputations participated in a randomized clinical trial comparing their prescribed, unpowered prosthesis and the BiOM powered prosthesis. Participants’ metabolic costs and self-selected walking speeds were measured in the laboratory and daily step count, daily steps away from home, and walking speed were measured over two weeks of at-home prosthesis use. Participants also rated their perception of mobility and quality of life and provided free-form feedback. Dependent measures were compared between prostheses and the relationships between metabolic cost, perception of mobility, and characteristics of walking in daily life were explored using Pearson’s correlations.

**Results:**

Twelve people were randomly allocated to the powered prosthesis first (n = 7) or unpowered prosthesis first (n = 5) and ten completed the full study. There were no differences in metabolic costs (p = 0.585), daily step count (p = 0.995), walking speed in-lab (p = 0.145) and in daily life (p = 0.226), or perception of mobility between prostheses (p ≥ 0.058). Changes varied across participants, however. There were several medium-sized effects for device comparisons. With the powered prosthesis, participants had increased self-reported ambulation (*g* = 0.682) and decreased frustration (*g* = 0.506).

**Conclusions:**

There were no universal benefits of the powered prosthesis on function in the lab or home environment. However, the effects were subject-specific, with some reporting preference for power and improved mobility, and some increasing their activity and decreasing their metabolic effort. Additionally, self-reported preferences did not often correlate with objective measures of function. This highlights the need for future clinical research to include both perception and objective measures to better inform prosthetic prescription.

*Trial registration:* https://clinicaltrials.gov, #NCT02828982. Registered 12 July 2016, https://clinicaltrials.gov/ct2/show/NCT02828982

**Supplementary Information:**

The online version contains supplementary material available at 10.1186/s12984-021-00842-2.

## Introduction

People with transtibial amputations (TTA) walk with greater asymmetry, using more metabolic energy, and prefer to walk more slowly than people without amputation [1]. These factors may contribute to their decreased physical activity level [2]. In particular, people with TTA have lower daily step counts [3] and walk for shorter durations at a time, compared to people without amputation [4, 5]. This deficit is important to address, as physical inactivity is related to lower quality of life [6] and can lead to secondary comorbidities such as obesity and cardiovascular disease [7].

Powered ankle-foot systems, such as the BiOM (now Ottobock Empower, Duderstadt, Germany) aim to reduce gait asymmetries and metabolic effort by providing active “ankle” power during the push-off phase of gait [8]. Prior studies have found that the powered prosthesis enabled people with TTA to use less metabolic effort to walk over level-ground [9, 10], while others found no differences on level-ground [11] or on slopes [12]. Similarly, while some studies found that participants walked at faster speeds with the powered prosthesis over a loose rock surface [13] and on level surfaces [9, 14], a more recent study found no differences in self-selected walking speed [11]. The participant cohort in the latter study differed from earlier ones as participants were older and potentially less physically active. Further, the study found that people designated as the highest Medicare functional classification level (K4) had reduced metabolic effort with the powered prosthesis, while those at a lower level (K3) did not [11]. This suggests that the benefits of prosthetic ankle power may depend on characteristics of the user, as the Medicare Functional Classification Level, or K-level, is a system that describes the rehabilitation potential of a person with lower-limb amputation [15].

While mixed, prior studies provide some evidence that prosthetic ankle power can be effectively incorporated into the user’s biomechanics to reduce their effort. It is unclear, however, whether this translates to changes in physical activity in daily life. Prior work has exclusively characterized measures of *capacity*, or what one is *capable of* in a standardized or optimal environment. According to the International Classification of Functioning, Disability and Health (ICF), evaluating *performance*, i.e., what one *does* in their actual environment, is also an important component of characterizing functionality [16]. Because the patient’s surroundings can play a large role in the accessibility to physical activity, it is imperative that evaluations of physical activity are made in the patient’s everyday environment. As such, a growing number of studies have employed community-based activity monitoring to evaluate prosthetic interventions, to provide clinicians with more comprehensive characterizations of the patient’s functional mobility [17]. The ICF also recommends that to properly measure improvements in health, psychological and social aspects of health should also be collected, which may heavily impact everyday performance. Overall, there are numerous factors that can contribute to or limit a patient’s performance in everyday life. Evaluating changes to those factors is a necessary step in moving toward a more comprehensive evaluation of powered prostheses.

Examining patient perception is one way to track changes to psychosocial factors that may affect mobility. While metabolic effort undoubtedly represents valuable information, it may not necessarily correlate to the patient’s perception of exertion [18]. Further, a previous study found no statistical difference in participants’ Prosthesis Evaluation Questionnaire scores between using unpowered and powered prostheses [14], whereas the same cohort reduced their metabolic costs with the powered prosthesis [10]. Given these differences, it is important to explore both perception of effort and measures of effort as is it unclear which relates more to a person’s physical activity. For example, if a device is perceived to be easier to walk with, even if it does not objectively reduce metabolic effort, this may alleviate conscious barriers to physical activity and enable an increase in the amount of physical activity.

In this study, we conducted a randomized crossover trial comparing the use of unpowered and powered prostheses in people with TTA, after one week of unsupervised device acclimation. Our primary goal was to quantify differences in metabolic cost, the volume (step count) and characteristics (walking speed) of everyday walking, as well as patient perceptions of their mobility and quality of life when wearing each prosthesis. We hypothesized that there would be differences in metabolic cost, step count, and walking speed when using the powered prosthesis, compared to the unpowered prosthesis. Based on prior work, we also hypothesized that participants would not perceive a change in mobility with the powered prosthesis. A secondary aim of this work was to explore the relationship between patient perceptions and functional outcomes measured in the lab and in daily life.

## Methods

### Participant recruitment

People with unilateral transtibial amputations (TTA) were recruited through clinical referral from the University of Michigan Orthotics and Prosthetics Center and through flyers and postings on umhealthresearch.org and clinicaltrials.gov (NCT02828982). Inclusion criteria included: aged 21 years or older, unilateral TTA, and prosthesis use for at least six months. Potential participants were excluded if they had a history of cardiovascular disease, or orthopedic or neurological disorders to their intact limb, or were unable to walk independently for 10 minutes at a time. Participants’ K-levels were obtained from their physician. We initially recruited older community ambulators (K3) who may be less physically active than in previous works [10, 14], as benefits of the powered prosthesis were less clear for this population [11]. However, due to recruiting difficulties, we later included more active community ambulators (K4). All participants provided their written informed consent prior to participation.

### Experimental protocol

This study utilized a cross-over design where participants were randomly assigned to perform testing first with their prescribed, unpowered prosthesis or with a powered prosthesis. For the powered condition, a certified prosthetist fit participants with a BiOM T2 powered prosthesis (BionX Medical Technologies Inc., Bedford, MA, USA) and tuned the device according to procedures described in Gardinier et al. [11]. They were then given one week to acclimate to the device at home and did not receive any device-specific training. Participants returned to the clinic if they needed any adjustments to their prosthetic settings or alignment. After any change, participants were given another week to acclimate. Only two participants (S03, S06) required readjustments. For the unpowered condition, participants needed to be stable in their prescribed prosthesis (no adjustments) for a period of at least one month prior to collection.

After the acclimation period, participants were given two activity monitors (ActiGraph GT9X Link, ActiGraph, Pensacola, FL, USA) and a global positioning system (GPS) enabled smartphone for a two-week period. One activity monitor was mounted on top of the prosthetic foot and collected accelerometer and gyroscope (IMU) data at 100 Hz, while the other (ACC) was attached to the lateral side of the prosthetic pylon and collected accelerometer data at 30 Hz. The placement for the ACC was chosen for its high test-retest reliability for step counts [19], while the IMU was place on top of the foot to ensure minimum movement during foot-flat [20]. GPS data were collected using either Ethica (Ethica Data, Ontario, Canada) or MapMyRun (Under Armour, Baltimore, MD). Participants were given an activity log to record their activity during the collection.

Following acclimation and activity data collection (≥ 3 weeks), participants came to the lab for metabolic testing and to complete questionnaires assessing their overall health and quality of life. Participants were instructed to fast for at least four hours prior to metabolic testing. We used a portable metabolic system (Cosmed K4b^2^, Rome, Italy) to measure participants’ oxygen consumption and carbon dioxide production. We first measured baseline metabolic costs as participants rested in a seated position for at least 10 minutes. We then measured metabolic costs while participants walked on a treadmill at a controlled speed based on leg length [21]. Participants walked for a minimum of three minutes after they achieved steady-state oxygen consumption, characterized by a visible plateau [22]. Once participants felt rested, we measured their self-selected walking speed by having them walk over a straight 8 m walkway, ten times.

Participants completed the Prosthesis Evaluation Questionnaire (PEQ) and Short Form (SF)-36 after each prosthetic condition and a Prosthesis Preference questionnaire at the end of the study. The PEQ consists of 82 questions that describe the function of a lower-limb prosthesis and assesses prosthesis-related quality of life [23]. The questionnaire is divided into ten functional scales, addressing four major domains: prosthetic function, mobility, psychosocial experience, and well-being. Participant’s quality of life was assessed using the Short Form (SF)-36 general health questionnaire, which provides eight component scores and Physical and Mental component scores. The Prosthesis Preference Questionnaire consisted of a single question where participants were asked to indicate which device they preferred on a 100 mm visual analog scale (VAS) from their unpowered device (0) to the powered ankle (100). Finally, using a semi-structured questionnaire, we asked participants for subjective feedback about their likes and dislikes and what if anything felt easier and/or harder with the powered prosthesis. If not mentioned, we then specifically asked about the ease of walking faster, longer, and on different types of terrain (e.g. uneven ground, stairs, slopes).

### Data analysis

We first verified that the last three minutes of breath measurements were at a steady state by confirming a respiratory exchange ratio between 0.7 and 1.0. Using the recorded steady-state oxygen consumption and carbon dioxide production rates, we estimated energy expenditure using the Brockway equation [24]. To generalize energy expenditure across participants, we calculated a dimensionless metabolic cost of transport (COT) by normalizing energy expenditure by participant weight and walking speed [25].

The accelerometer and IMU were programmed to begin data collection on the day following meeting with study personnel at 12 am, to avoid partial day collections. The accelerometer collected data until the battery died, which was typically around 12 days. We excluded data from days in which wear time was < 6 hours [26], which may be due to participants not wearing the prosthesis or leaving the accelerometer on the charger for the day. ActiLife software (ActiGraph, Pensacola, FL, USA) calculated daily step counts as double the single leg stride count from the pylon-mounted accelerometer. Periods of non-sedentary activity were defined as any period of activity greater than 30 counts per minute epoch [27]. Such a low threshold includes small movements and detects even a single stride as ‘non-sedentary activity.’ We then separated steps that occurred during the active period as ‘at home’ and ‘away from home’ using the time matched GPS data [26].

Because the pylon-mounted accelerometer recorded data at a low sampling rate (30 Hz), battery life lasted approximately 12 days, and we did not instruct participants to recharge the accelerometer overnight. IMU battery life was typically only 24 hours, due to the higher sampling rate. As such, participants were instructed to charge the IMU every night. Because we did not derive daily averages from the IMU, we did not exclude data from days with low wear time. To calculate stride-by-stride walking speed, we first calculated the position trajectory of the prosthesis-mounted IMU using a strapdown inertial navigation algorithm [28]. Briefly, the algorithm integrated the acceleration signal twice to calculate a position trajectory and applied zero velocity updates at every foot-flat to reduce drift error [20]. Strides were segmented with heel strikes, detected using the acceleration signal (vertical peak acceleration > 6 G and 500 ms between peaks) and velocity estimates (5 ms-window mean vertical velocity < 0). We then used this data to calculate walking speed according to methods described in Kim et al. [26].

For the PEQ and SF-36, we averaged scores for questions within each domain or sub-scale, omitting any blank entries. Values were only included where participants answered more than 50 % of the questions in that domain [23].

### Statistical analysis

To mitigate the effects of varying amounts of accelerometer and IMU data collected by each participant, we calculated the bootstrapped mean for each outcome measure taken during everyday activity (step counts, walking speed). For example, from a data set with size *n*, we sampled *n* elements with replacement, took the mean of the resampled set, and repeated this process 1000 times. The mean of all 1000 resampled means is the bootstrapped mean [29], which was used for analysis. Self-selected walking speed in the lab was the average of 10 trials. We tested for differences in COT, daily step count, daily step count away from home, walking speed (in-lab and in daily life), PEQ (by sub-scale), and SF-36 (by sub-scale and physical and mental components) between the two prostheses (unpowered, powered) using a series of paired t-tests. We assessed whether prosthesis preference was significantly different from 50 (no preference) using a one sample t-test. Significance was set to p < 0.05 for all comparisons. Given the small sample size, we calculated the effect sizes for all pairwise comparisons using Hedge’s *g*:

$$g=\frac{{M}_{Powered}-{M}_{Unpowered}}{{SD}_{pooled}}\times \frac{N-3}{N-2.25}\times \sqrt{\frac{N-2}{N}}$$

$${SD}_{pooled}=\sqrt{\frac{\left({{SD}_{Powered}}^{2}\left({n}_{Powered}-1\right)\right)+\left({{SD}_{Unpowered}}^{2}\left({n}_{Unpowered}-1\right)\right)}{{n}_{Powered}+{n}_{Unpowered}-2}}$$

where *M*_*x*_ is the mean of group *x*, *SD*_*poole*d_ is the pooled standard deviation, *N* is the sample size, *SD*_*x*_ is the standard deviation within group *x*, and *n*_*x*_ is the sample size of group *x* [30]. Effect sizes are interpreted as being small for 0.2 ≤ *g* ≤ 0.5, medium for 0.5 ≤ *g* ≤ 0.8, and large for *g* ≥ 0.8 [31]. We also explored the relationships between COT, activity during daily life, prosthesis preference, and patient perception using Pearson correlations. We noted comparisons with medium effect sizes (|*g*| ≥ 0.5) in the results section.

## Results

### Participant details

**Fig. 1:**
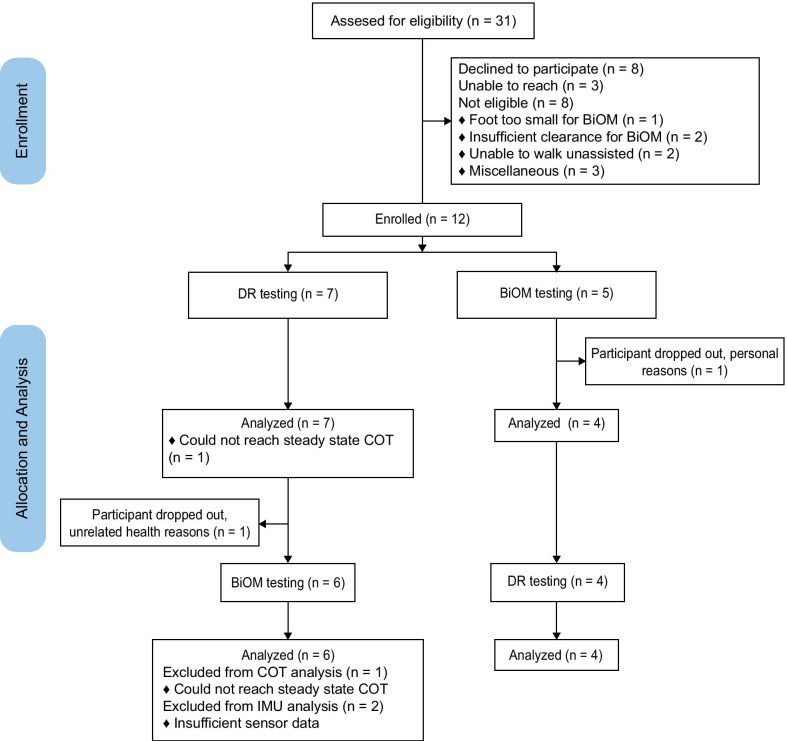
Consort flow diagram illustrating recruitment, enrollment, exclusion, and analysis

A total of 31 patients were contacted about the study (Fig. 1). Eight declined to participate citing the time commitment or their lack of interest in new prostheses, while three others did not respond. Another eight were deemed ineligible. The remaining 12 individuals were randomly allocated to a prosthetic testing order (Table 1). S09 dropped out before completing the study due to an unrelated medical condition. S10 was assigned to the powered prosthesis first, acclimated to the device and was provided activity monitors. During this time, he developed a wound on his residual limb and subsequently dropped out of the study. Ten males (52.6 ± 11.3 years old) completed the study. Nine were classified as K3 and one was classified as K4 on the Medicare K-level.

**Table 1:** Participant demographics

ID	Age (years)	Sex	Mass (kg)	Height (m)	Cause of amputation	Non–Powered Prosthetic Foot	K–Level	Time Since Amputation	Completed Full Study?
S01	57	M	98.4	1.85	Vascular	DR	K3	3 years	Y
S02	62	M	118.8	1.82	Trauma	DR	K3	3 years	Y
S03	53	M	128.0	1.78	Vascular	DR	K3	8 years	Y
S04	65	M	84.1	1.82	Vascular	DR	K3	3 years	Y
S05	40	M	123.4	1.78	Trauma	DR	K3	12 years	Y
S06	30	M	90.8	1.80	Trauma	Hydraulic	K3	1.2 years	Y
S07	65	M	89.1	1.64	Trauma	Hydraulic	K3	9 years	Y
S08	55	M	108.0	1.83	Trauma	DR	K3	1.2 years	Y
S09	27	M	73.0	1.89	Trauma	DR	K4	5 years	N
S10	–	M	71.7	1.72	Trauma	DR	–	–	N
S11	54	M	83.0	1.77	Trauma	DR	K4	19 years	Y
S12	45	M	98.4	1.85	Trauma	DR	K3	5.5 years	Y

DR is a dynamic response foot

### Metabolic cost

There were no differences between the fixed treadmill speed (1.20 ± 0.07 m/s) and self-selected walking speeds with the unpowered (1.16 ± 0.16 m/s; p = 0.435) or powered prostheses (1.21 ± 0.12 m/s; p = 0.794). S03 was not able to achieve steady-state energetic expenditure on a treadmill. For the remaining participants, there was no group difference in metabolic cost of transport (COT) between prostheses (n = 9; p = 0.585, *g* = -0.150), but there was variability across participants. While six participants had lower COT with the powered prosthesis, only two participants had reductions greater than the between-day minimal detectable change of 0.051 J/Nm [22].

**Table 2:** Step count and walking speed in-lab and in daily life (mean ± SD)

	Unpowered	Powered	*p*–value	Hedge’s *g*
Daily step count, overall	4770 ± 2150	4760 ± 2150	0.995	− 0.001
Daily step count away from home	2030 ± 1440	1640 ± 1100	0.452	− 0.248
Walking speed, in–lab (m/s)	1.15 ± 0.16	1.18 ± 0.16	0.145	0.310
Walking speed, overall (m/s)	0.76 ± 0.10	0.74 ± 0.10	0.226	− 0.158

**Fig. 2:**
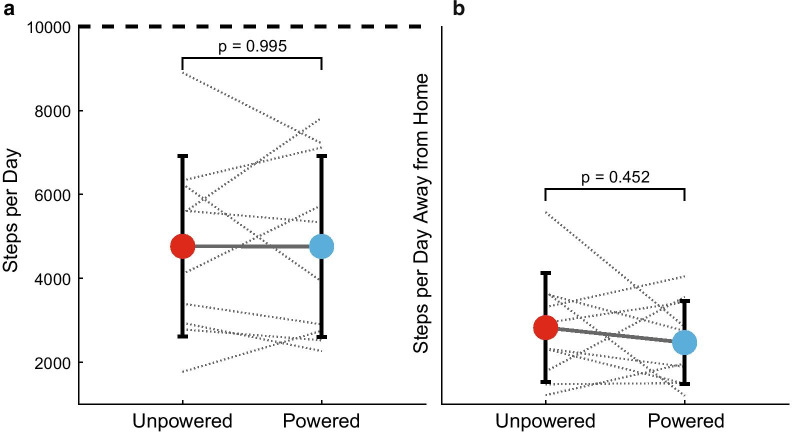
**a** Daily step count using the unpowered (red) and powered (blue) prostheses. Dashed horizontal line represents the recommended 10,000 steps per day. **b** Daily step count away from home using the unpowered (red) and powered (blue) prostheses. Gray x’s and lines represent individual participant trends

### Activity data

There were no differences in the bootstrapped daily step count (p = 0.995, *g* = − 0.001; Fig. 2a) or daily step count away from home (p = 0.452, *g* = − 0.248; Fig. 2b) between prostheses (Table 2). While step counts varied across participants, none achieved the recommended 10,000 steps per day [32].

**Fig. 3:**
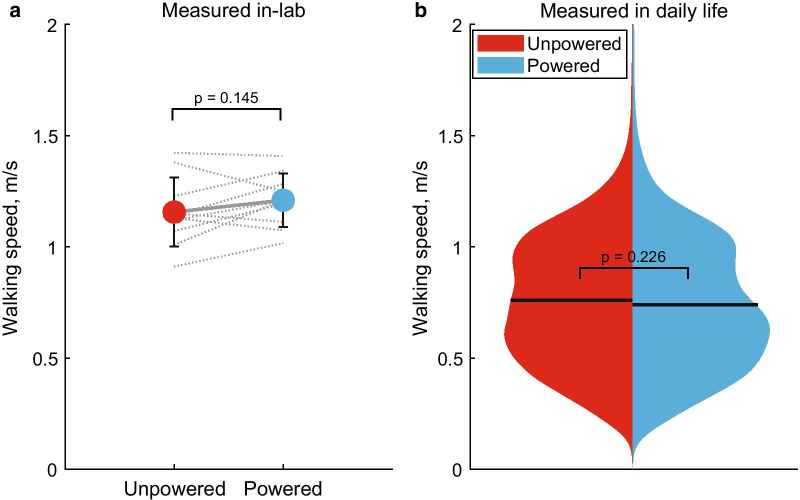
**a** Self-selected walking speeds measured in the lab for participants using the unpowered (red) and powered (blue) prostheses. Gray x’s and lines represent individual participant trends. **b** Split violin plot of the probability density functions of walking speed distributions of walking strides taken in daily life. To visualize, raw distributions were smoothed using the *ksdensity* kernel smoothing function in MATLAB. Shared regions are averaged distributions and solid horizontal lines are the group means

There was no difference between prostheses in self-selected walking speeds in the lab (p = 0.145, *g* = 0.310; Fig. 3a). Though S05 and S06 completed the study, due to a sensor malfunction, the activity monitor did not record sufficient IMU data with the powered prosthesis. These participants were therefore excluded from all daily-life walking speed comparisons. There was no difference between prostheses in walking speeds during daily life (n = 8; p = 0.226, *g* = − 0.158; Fig. 3b).

### Questionnaires

**Fig. 4:**
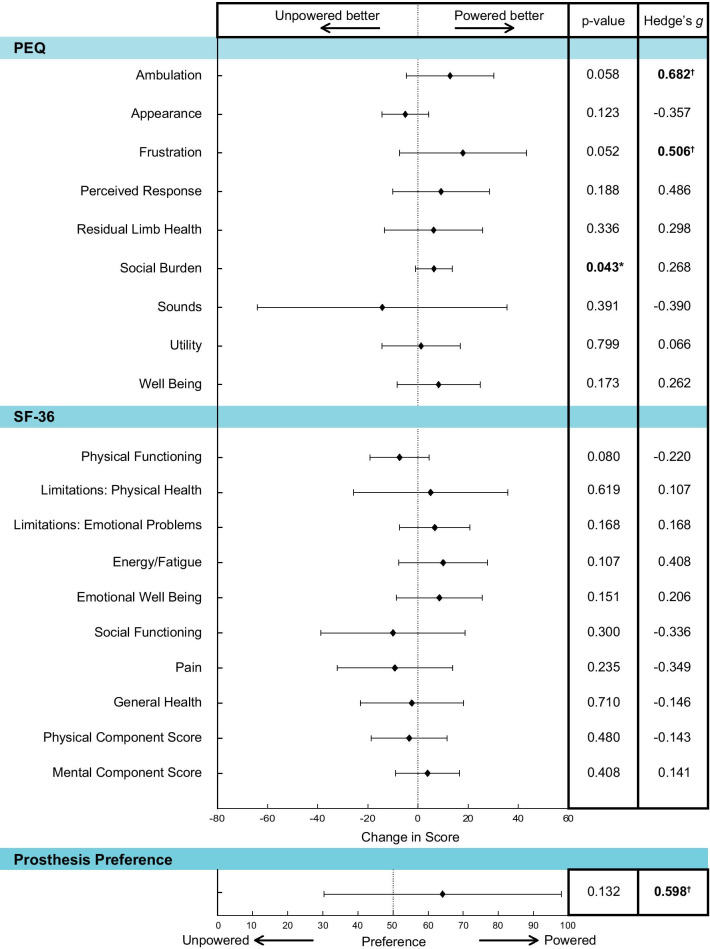
Changes in participant responses for the Prosthesis Evaluation Questionnaire, Short Form-36 and Prosthesis Preference, by sub-scale. Significant differences (p < 0.05) between prosthesis are indicated and bolded (*). Changes with a medium or large effect size (*g* ≥ 0.5) are also indicated and bolded (†)

In the Prosthesis Evaluation Questionnaire (PEQ), participants reported significantly less social burden with the powered prosthesis, compared to that of the unpowered prosthesis (p = 0.043, *g* = 0.268; Fig. 4). There were also non-significant, medium- and large-sized effects in the mobility and frustration sub-scales where participants reported better mobility (p = 0.058, *g* = 0.682) and less frustration (p = 0.052, *g* = 0.506) with the powered prosthesis. For the Short Form (SF)-36 questionnaire, there were no differences in the physical (p = 0.480, *g* = − 0.143) or mental component scores (p = 0.370, *g* = 0.141), or any of the individual sub-scales (p ≥ 0.080, |g| ≤ 0.408). While participants generally preferred the powered prosthesis (prosthesis preference score = 64.1 ± 33.8; *g* = 0.598), this was not significantly different from no preference(p = 0.132).

### User feedback

The open-ended user feedback was mixed across participants. One participant reported that they *liked* that the BiOM “almost felt like a real ankle” while another participant similarly commented that walking with the BiOM “felt more natural.” Six participants *disliked* that the BiOM batteries die too soon, two said it was too bulky, one said it was too noisy, one said it was too heavy, and one described the BiOM as being too controlling and causing more phantom pain. Six participants felt that they could walk *faster* with the BiOM, while four did not. Five participants felt they could walk for *longer* when wearing the BiOM. Five participants found walking to be *easier*, five found it easier to walk on slopes, six found it easier to walk upstairs, and three found it easier to walk downstairs. In contrast, four found level-ground walking *harder*, five found walking down stairs to be more difficult, four found uneven terrain (specifically grass and snow) to be more difficult, two found driving more difficult and one found it more difficult to stand from a chair. The remaining respondents did not notice a difference or did not perform that activity.

**Fig. 5:**
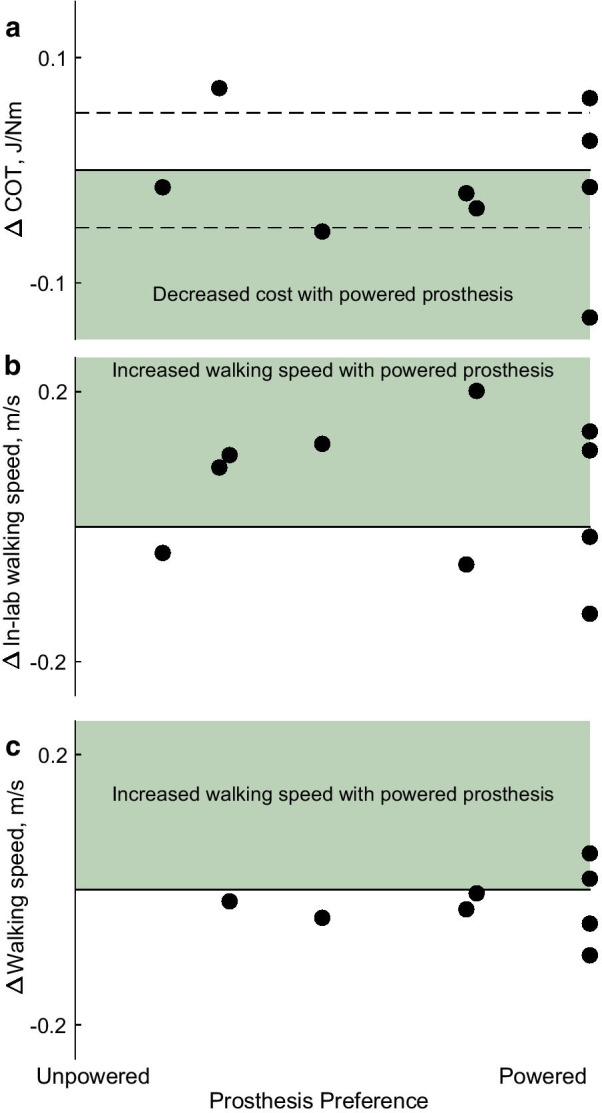
**a** Relationship between changes in prosthesis preference and changes in metabolic cost (ΔCOT). Dashed lines indicate the minimal detectable change in COT. **b** Relationship between changes in prosthesis preference and changes in daily step count. **c** Relationship between changes in prosthesis preference and changes in walking speed in daily life. Linear fits are shown for moderate correlations (r ≥ 0.6)

### Correlations

Prosthesis preference was not correlated with changes in COT (r = − 0.181, p = 0.667; Fig. 5a), with changes in walking speed measured in-lab (r = − 0.086, p = 0.814; Fig. 5b), or with changes in walking speed in daily life (r = 0.070, p = 0.869; Fig. 5c).

**Fig. 6:**
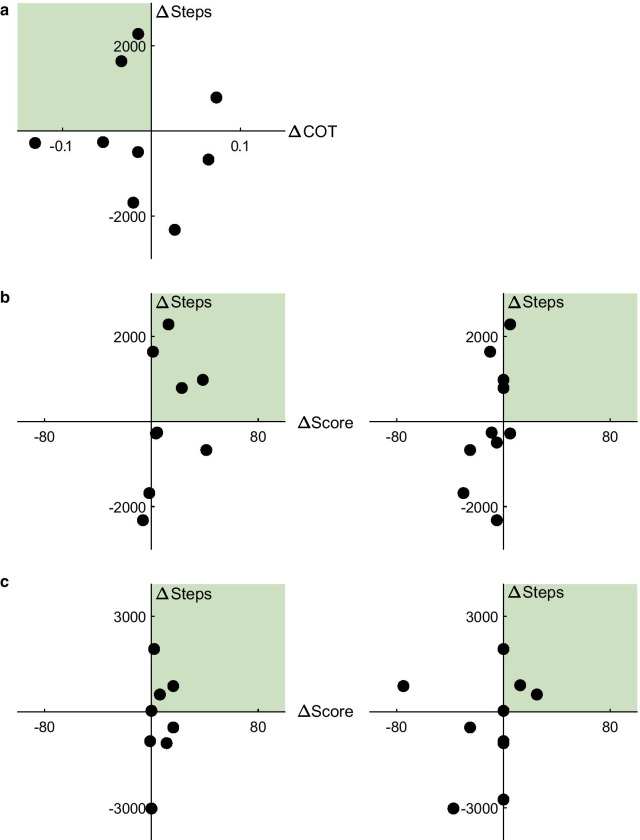
**a** Relationship between changes in metabolic cost (ΔCOT) and changes in daily step count. Data in the second quadrant (highlighted in green) indicate lower metabolic cost and greater step count with the powered prosthesis. **b** Relationships between changes in the PEQ ambulation sub-scale (left) and the SF-36 physical functioning sub-scale scores (right) and changes in daily step count.**c** Relationships between changes in the PEQ social burden sub-scale (left) and the SF-36 social functioning sub-scale scores (right) and changes in daily step count away from home. Data in the first quadrant (highlighted in green) indicate greater scores and greater step count with the powered prosthesis

Changes in daily step count were not correlated with changes in COT (r = − 0.074, p = 0.849; Fig. 6a), or perception of mobility when assessed with the PEQ ambulation sub-scale (r = 0.324, p = 0.395; Fig. 6b). There was a moderate correlation between changes in step counts and the SF-36 physical functioning sub-scale (r = 0.505, p = 0.137; Fig. 6b). There were no relationships between changes in step count away from home and the PEQ social burden sub-scale (r = 0.204, p = 0.628) or the SF-36 social functioning sub-scale (r = 0.120, p = 0.740; Fig. 1c).

## Discussion

Contrary to our hypothesis, we did not find differences between prostheses in metabolic costs. Differences in metabolic cost between prostheses varied across participants, however. Two participants reduced their metabolic effort by more than the minimum detectable change (MDC) of 0.051 J/Nm, while two others increased their metabolic cost more than this amount [22]. These findings agree with one previous study [11], but disagree with two others [9, 10]. There are two notable differences in these prior works. In those studies that found a metabolic benefit, participants were young or physically active and had time to adjust to the powered prosthesis (≥ 2 hours). The study that did not find a benefit tested an older, less active cohort and only provided a short time (~ 15 min) for device accommodation. Here, we also tested a population that was generally less active, but participants had a minimum of three weeks of device use prior to metabolic testing. Further, two participants already owned the BiOM and had been regularly using the device for at least 6 months. The two BiOM owners had contrasting responses to the powered prosthesis in metabolic cost, step count, and walking speed. While this may suggest that the lack of metabolic benefit is more related to patient characteristics than accommodation time, it is also possible that less active individuals require even more accommodation or more focused rehabilitation. Although there is no consensus on the time required to acclimate to a prosthetic intervention [33], the accommodation provided here falls in line with previous studies that found significant metabolic reductions after 1.5 weeks [34] and 21 days [35] of a prosthetic intervention. There is also no set training for adjusting to a new prosthesis. This likely contributes to the variability in participant responses. Our user feedback on learning to use the powered prosthesis also supports this idea as some felt they learned “right away” while others said they “still haven’t figured it out yet” (Additional file 2). Additionally, while we ensured a minimum time period for acclimation, we could not control for the actual amount of acclimation, as this may depend on how much each participant used the prosthesis in daily life. As seen in daily step counts, this varied widely among participants (Fig. 3). Thus, we should potentially view accommodation as a function of steps taken, rather than in days of use.

Given the varied metabolic responses to the powered prosthesis, we also explored if users perceived walking to be easier with the powered prosthesis. Five participants felt that walking with the powered prosthesis was easier while four participants responded that walking with the powered prosthesis was harder. Of the five participants that felt walking with the powered prosthesis was easier, only one had reduced metabolic cost with the powered prosthesis, while another had greater metabolic cost (Table 3). Similarly, of the four participants that felt walking with the powered prosthesis was harder, one had increased metabolic cost, while another had decreased metabolic cost with the powered prosthesis. This agrees with prior work that found that the perception of exertion contrasts to the physiological measure of metabolic cost [18].

**Table 3:** Self-reported perception of mobility and changes in capacity and performance

	User feedback regarding the powered prosthesis			Changes in outcome measures (+: increase from unpowered to powered)			
ID	Is walking easier/harder? (including uneven terrains)	Can walk faster?	Can walk for longer?	Metabolic cost	Daily step count	Walking speed, in–lab	Walking speed, daily life
S01	Easier	Y	Y	− 0.131^a^	− 280	0.14^b^	− 0.05
S02	Easier	Y	Y	− 0.015	2280	− 0.02	− 0.10
S03	Harder	N/A	N	N/A	980	0.11	− 0.02
S04	Easier	N/A	Y	0.026	− 2320	− 0.13^b^	0.05
S05	Harder	N/A	N	0.073^a^	790	0.09	N/A
S06	Harder	Y	N/A	− 0.015	− 500	− 0.04	N/A
S07	Harder	Y	N/A	− 0.054^a^	− 260	0.12^b^	− 0.04
S08	N/A	N/A	Y	− 0.034	1640	0.20^b^	− 0.01
S11	Easier	Y	Y	0.064^a^	− 670	0.11^b^	0.02
S12	Easier	Y	N	− 0.020	− 1680	− 0.06	− 0.03

^a^Changes in metabolic cost greater than between-day minimal detectable change of 0.051 J/Nm

^b^Changes in walking speed greater than minimal detectable change of 0.108 m/s

Further, we explored how everyday physical activity levels might reflect changes in metabolic costs or perceived ease of walking. Among the five participants that felt walking with the powered prosthesis was easier, only one increased their daily step count (Table 3). Physical activity in daily life may be more dependent on factors other than the prosthesis, such as the surrounding environment, weather, lifestyle, personality, and occupation. In particular, walking with the powered prosthesis is more destabilizing when walking on icy or otherwise slippery surfaces, which may have influenced participants’ walking patterns and confounded our results. Though we could not control the weather conditions, we did collect each participants’ activity with both prostheses in a single season, when possible (see Additional file 1). Similar to our findings, a previous study evaluating the effects of a microprocessor knee found no differences in everyday activity [4].

Participants also had varied feelings about the powered prosthesis and how it improved or did not improve their function. Four preferred their prescribed, unpowered prosthesis while six preferred the powered prosthesis. Prosthesis preference was not related to changes in metabolic cost or walking speed. While there was no relationship between prosthesis preference and measures of functional capacity or performance, participants who preferred the powered prosthesis tended to feel that the powered device helped them walk for longer without rest, faster (Additional file 2), and with more ease (Table 3). This user feedback may provide information regarding the factors that determine prosthesis preference and/or acceptance.

There were differences between participants’ perception of their function and their performance in daily life. While six participants responded that they felt they could walk faster with the powered prosthesis, only three walked faster in-lab by more than 0.108 m/s (MDC for older adults in 4-meter walk tests) [36], and only one walked faster in daily life, by an amount far less than the in-lab MDC of walking speed (Table 3). Furthermore, while five participants responded that they felt they could walk for longer with the powered prosthesis, only two participants increased their daily step count. Comparing qualitative user feedback and measures of step count and walking speed in daily life, there seemed to be a disconnect between what people perceived they were capable of doing and what they did in daily life. This dissonance is supported by the weak correlations between changes in step count and changes in the PEQ ambulation sub-scale and changes in the SF-36 physical functioning sub-scale. This suggests that future research and clinical approaches to prescription should consider both perception and objective measures. This is important as daily prosthesis use is largely dependent on an individual’s feelings about their function, while device prescription is predominantly supported by more objective measurable outcomes.

Psycho-social responses to the powered prosthesis may affect physical activity, especially in community settings. Participants perceiving less social burden with the powered prosthesis contrasted with previous findings with a younger cohort [14], which suggests that psycho-social responses may be age-dependent. This may be attributed to the higher likelihood for older individuals to be in co-dependent domestic relationships, as the social burden sub-scale describes one’s perception of how the prosthesis affects the relationships with their partner or family members [23]. However, the weak correlations between changes in community engagement and changes in psycho-social sub-scales of the PEQ and SF-36 suggest that other factors may influence community engagement more strongly. A more practical limiting factor for community engagement may be the short battery life, as expressed in user feedback by six participants. Because the heavy weight of the powered prosthesis is more noticeable when the battery dies and makes walking harder, users may choose to engage in the community only when they are equipped with several fully charged batteries. The Ottobock Empower (current version of the BiOM) has a battery life of 8 hours, which may alleviate these issues. However, battery life is dependent on intensity of use and may still be a concern for very active individuals who would require a battery change for all-day use.

This study had several limitations. Walking speed in daily life was calculated from all straight-line over-ground walking strides, which had variable sample sizes as participants did not all log the same number of strides in daily life. We addressed this issue by calculating the bootstrapped mean of walking speeds, thereby minimizing bias caused by varied sample sizes. Further, consistent with previous studies done in the lab [9–11], we focused only on straight-line strides and thus did not include turning strides or stair-walking. While more work can be done toward specifically identifying and examining non-straight-line walking, this may require additional sensors on the hip or intact foot. We chose to only attach the sensors directly on the prosthetic foot to minimize the day-to-day variability in sensor placement and maximize sensor wear time. Further, data for the powered prosthesis may include steps for which the person did not receive power, either because the battery died or because the participant turned it off (e.g., to traverse uneven terrain). We cannot identify these steps based on the data from our sensors. Based on participant feedback, however, we expect that theses instances represent a very small portion of measured steps. As mentioned above, weather conditions may have also affected everyday performance. While collections for different prostheses were done mostly in the same season, one participant’s everyday activity was collected in different seasons due to scheduling conflicts (see Additional file 1). Additionally, some variability in performance may be due to lifestyle or life events (e.g., vacation, hospitalization), rather than the prosthesis. No participants reported such events in their activity logs, though several participants were retired and did not have a regular day-to-day schedule. Lastly, this study was limited by a small sample size due to difficulties in recruitment. To mitigate these difficulties, we amended the study to additionally recruit K4 participants, which further diversified the already heterogenous cohort of K3 participants. The low sample size increases the likelihood of type II errors. To address this issue, we have provided effect sizes for all comparisons. Future studies should confirm these findings in larger cohorts.

## Conclusions

This study compared participants’ metabolic costs, walking speeds in-lab and in daily life, step count, step count away from home, perceived mobility, and preference between powered and unpowered prostheses. There was no statistically significant preference for either prosthesis. Additionally, wearing the powered prosthesis did not significantly decrease metabolic costs, increase physical activity or walking speed, or increase perceived mobility. Though the powered prosthesis was not universally beneficial to the participant cohort, the large variability in responses across participants suggests that different people may benefit in different ways and to varying degrees. Regarding the powered prosthesis, participants reported feeling they could walk faster and with more ease, while battery life and weight were prevalent complaints. There was disparity between participants’ perceptions of their mobility and what they perform during daily life when using the powered prosthesis. This suggests that future research should continue to examine both perception and objective measures of mobility to better evaluate prostheses and inform prescriptions of advanced prosthetic components.

## Supplementary Information

**Additional file 1.** Accelerometer and IMU data collection months, number of days recorded, and average wear time per day for each participant.

**Additional file 2.**  Participants’ individual responses to the semi-structured survey and their relative preference for the unpowered or powered prostheses.

## Data Availability

The datasets used and/or analyzed during the current study are available from the corresponding author on reasonable request.
