# Supplementary material for: The influence of powered prostheses on user perspectives, metabolics, and activity: a randomized crossover trial
Source: J Neuroeng Rehabil. 2021 Mar 16;18:49. doi: 10.1186/s12984-021-00842-2 (PMC7962267; doi:10.1186/s12984-021-00842-2)
Supplement: Supplementary file 1 — Additional file 1. Accelerometer and IMU data collection months, number of days recorded, and average wear time per day for each participant. [file 12984_2021_842_MOESM1_ESM.pdf]

Additional File 1a. Accelerometer data collection months, number of days recorded, and average wear time per day for each participant

ID	Unpowered			Powered		
	Months	# Days	Wear time / day (hrs)	Months	# Days	Wear time / day (hrs)
S01	Jul – Aug	13	14.6	Aug	13	14.4
S02	Sep – Oct	18	16.1	Oct – Nov	21	17.0
S03	Dec – Jan	13	10.9	Jan – Feb	11	12.3
S04	Mar – Apr	14	16.3	Apr – May	15	15.4
S05	Aug – Sep	11	13.4	Jul – Aug	11	14.4
S06	Sep – Oct	12	7.3	Nov – Dec	10	4.4
S07	Mar – Apr	29	9.3	May	13	9.6
S08	Feb	8	14.1	Aug	9	14.6
S11	Apr – May	27	13.9	Mar – Apr	25	9.9
S12	May – Jun	19	7.0	Apr – May	28	10.6
**Mean**		16.4	12.3		15.6	12.3
**SD**		6.9	3.4		6.7	3.7

Additional File 1b. IMU (Accelerometer & gyroscope) data collection months, number of days recorded, and average wear time per day for each participant

ID	Unpowered			Powered		
	Months	# Days	Wear time / day (hrs)	Months	# Days	Wear time / day (hrs)
S01	Jul – Aug	5	12.0	Aug	1	8.6
S02	Sep – Oct	8	9.2	Oct – Nov	18	12.5
S03	Dec – Jan	6	10.9	Jan – Feb	1	8.0
S04	Mar – Apr	2	11.1	Apr – May	5	11.3
S05	Aug – Sep	1	12.9	Jul – Aug	1	1.7*
S06	Sep – Oct	4	3.7	Nov – Dec	1	0.0*
S07	Mar – Apr	20	9.1	May	30	9.5
S08	Feb	8	13.9	Aug	6	15.4
S11	Apr – May	14	11.2	Mar – Apr	8	10.6
S12	May – Jun	7	9.2	Apr – May	12	9.1
**Mean**		7.5	10.3		8.3	8.7
**SD**		5.7	2.8		9.5	4.7

*IMU data was not sufficient so S05 and S06 were excluded from walking speed analysis in daily life
