# Supplementary material for: The influence of powered prostheses on user perspectives, metabolics, and activity: a randomized crossover trial
Source: J Neuroeng Rehabil. 2021 Mar 16;18:49. doi: 10.1186/s12984-021-00842-2 (PMC7962267; doi:10.1186/s12984-021-00842-2)
Supplement: Supplementary file 2 — Additional file 2. Participants’ individual responses to the semi-structured survey and their relative preference for the unpowered or powered prostheses. [file 12984_2021_842_MOESM2_ESM.pdf]

Additional File 2. User feedback and preference

ID	Did the BiOM help you walk/work longer without rest?	Can you walk faster with the BiOM?	How long did it take to learn? Did you ever learn?	Other comments	Preference[a]
S01	Yes	Yes	Leg felt heavy the first few days; Noticed he got more power by extending the knee. Pointed to quads and said "when I contract these muscles, I get more power out of it"	Needed socks because device is heavier & causes more pistoning. Got distal cup to lift residual limb over bursa	100
S02	Yes	Yes	Not answered	Not answered	100
S03	No; back pain	Not answered	3-4 days	Doesn't feel "even." Started having back pain. Didn't like the lift because it caused pain on top of foot	30
S04	Yes	Not answered	Adjusted quickly: "right away"	A little off balance with alignment; got heel caught on back of stair; some stability issues	100
S05	No	No	Took a month to get used to. Felt it gets easier over time. Still has not quite figured it out yet.	Not answered	28
S06	Not answered	Yes (only on flat ground)	Not answered	The BiOM makes you do what it wants you to do instead of doing what you want it to do. BiOM doesn't adapt to terrain well.	17
S07	Not answered	Yes	About an hour but sometimes it surprises him	Not answered	48
S08	Yes	Not answered	Not answered; BiOM owner	Not answered	78
S11	Yes	Yes	Not answered; BiOM owner	Would never walk with unpowered foot unless BiOM was broken	100
S12	No; calf pressure	Yes	Not answered	More calf pressure with BiOM	76

[a] Preference scale: 0 = unpowered, 100 = powered
